# LPWAN and Embedded Machine Learning as Enablers for the Next Generation of Wearable Devices

**DOI:** 10.3390/s21155218

**Published:** 2021-07-31

**Authors:** Ramon Sanchez-Iborra

**Affiliations:** Department of Engineering and Applied Techniques, University Center of Defense at General Air Force Academy, Santiago de la Ribera, 30729 Murcia, Spain; ramon.sanchez@cud.upct.es

**Keywords:** wearables, TinyML, LPWAN, LoRAWAN, machine learning

## Abstract

The penetration of wearable devices in our daily lives is unstoppable. Although they are very popular, so far, these elements provide a limited range of services that are mostly focused on monitoring tasks such as fitness, activity, or health tracking. Besides, given their hardware and power constraints, wearable units are dependent on a master device, e.g., a smartphone, to make decisions or send the collected data to the cloud. However, a new wave of both communication and artificial intelligence (AI)-based technologies fuels the evolution of wearables to an upper level. Concretely, they are the low-power wide-area network (LPWAN) and tiny machine-learning (TinyML) technologies. This paper reviews and discusses these solutions, and explores the major implications and challenges of this technological transformation. Finally, the results of an experimental study are presented, analyzing (i) the long-range connectivity gained by a wearable device in a university campus scenario, thanks to the integration of LPWAN communications, and (ii) how complex the intelligence embedded in this wearable unit can be. This study shows the interesting characteristics brought by these state-of-the-art paradigms, concluding that a wide variety of novel services and applications will be supported by the next generation of wearables.

## 1. Introduction

The wearable industry has notably evolved during the last years. From the first devices, which could be worn as accessories, e.g., wristbands or glasses, or integrated within clothing, e.g., footwear sensors, currently novel solutions that are tattooed or implanted in the body are gaining momentum [1]. The importance of wearables in today’s modern societies is prominent, as they are capable of continuously monitoring a large range of parameters and provide useful information in real time. Moreover, they are able to trigger pre-configured alerts, in order to interact with the device’s carrier in a dynamic fashion. Nowadays, the most popular application of wearables is fitness tracking, although others that are closely related to the eHealth ecosystem are also gaining relevance [2].

Although the design of wearable’s hardware and software is progressively being improved, most of the wearable units in the market present the following common characteristic: they should be linked to a master device, usually a smartphone, in order to exploit their full potential. Thus, the wearable unit is a mere data collector and/or alerting device, with highly restricted processing and communication capabilities. The main reason for adopting this strategy is the stringent constraints of these tiny elements, in terms of hardware resources (processing power, memory, storage, etc.) and energy consumption, as they are normally powered by small batteries [3].

Nevertheless, a new range of solutions in two relevant areas for the further development of wearables has recently emerged. On the one hand, the low-power wide-area network (LPWAN) communication paradigm will permit long-range and energy-efficient direct connectivity of wearable units with the cloud. This communication model is currently being quickly developed, and different solutions and platforms are already available in the market [4]. They will permit wearables to be detached from the master device to exchange data with the internet, hence making them independent units from this perspective. Although LPWANs have been deeply studied during the last years [5], their integration within wearable devices has not been well explored yet.

On the other hand, providing wearable units with intelligence would be of great relevance, in order to convert them into truly autonomous devices that are capable of making smart decisions using their collected data. However, so far, the most popular artificial intelligence (AI) family of mechanisms, i.e., machine learning (ML), has required a great amount of resources to be executed. Fortunately, the recently arrived TinyML paradigm fills this gap, by enabling the conversion of powerful ML models aiming at being runnable by constrained processing units, such as microcontrollers [6]. Besides, other ML approaches following a distributed strategy such as federated learning (FL), enable ML model building in a cooperative way [7].

The principal aim of this work is to investigate the synergies between these state-of-the-art concepts under the umbrella of the wearable ecosystem. To this end, this article explores and discusses these communication and AI techniques, which are highly suitable to be adopted by the next generation of wearable devices, hence, paving the way for the development of innovative services. These services and applications are also identified, and their main challenges are examined. Finally, an experimental study is presented, in which (i) the long-range coverage provided to a wearable unit by a smart campus LPWAN deployment is studied, and (ii) the limits of this constrained device for running intelligent mechanisms are evaluated. 

The rest of the paper is organized as follows. Section 2 provides insights regarding the integration of the LPWAN, TinyML, and FL paradigms within the wearable ecosystem. The potential applications and challenges for next-generation wearables are explored in Section 3. A wearable device architecture and its implementation in presented is Section 4. Besides, the results of a real case study, aimed at evaluating the LPWAN-based connectivity gained by a wearable device, as well as the AI complexity supported by this unit, are also presented in this section. The paper is concluded in Section 5, which also presents future research lines.

## 2. Background: Enabling Technologies

The Internet of Things (IoT) and AI paradigms have been a great revolution for the digitalization of many vertical industries. Nowadays, they have evolved enough to be adopted by the wearable industry, aiming at producing truly autonomous and intelligent devices. In the following, the communication and AI technologies that will drive the development of the next generation of wearables are examined.

### 2.1. Communications: Low-Power Long-Range Transmissions

The development of transmission technologies permitting long-distance communications with a highly limited energy consumption, has been one of the great achievements in the IoT ecosystem during the recent years [8]. This family of technologies, so-called LPWAN, is receiving great attention, given the almost omnipresence of connected devices in different scenarios, namely, urban, rural, aerial, etc., where a pre-existent communication infrastructure is not always present.

LPWANs bring interesting advantages for wearables over other traditional solutions that are traditionally employed in IoT systems, such as cellular communications, WiFi, Bluetooth low energy (BLE), or IEEE 802.15.4-based protocols (Zigbee or 6LoWPAN). Essentially, LPWAN-based systems adopt the typical cellular architecture, in which end-devices directly communicate with a central gateway or base station, in this case, within the ranges of 10 and 20 km in urban and rural scenarios, respectively [5]. As can be observed, this performance is greatly superior to those of the other mentioned technologies. These transmission distances are achieved with very low-power consumption, which is comparable to that of wireless sensor network (WSN) technologies, such as Zigbee or 6LoWPAN. Furthermore, the network architecture of LPWANs permits great scalability and node density, which is an important aspect in ultra-dense scenarios, such as highly populated cities. The network complexity of LPWANs is very low because, although possible [9], the cooperation among end-devices is not required to reach the central point, so neither routing protocols nor node coordination are needed in this type of deployment.

These interesting characteristics are achieved thanks to a series of strategies. Firstly, LPWAN solutions make use of highly reliable modulation and coding schemes. They permit to send data at very long distances, with a very low-power transmission, at the time of being greatly robust to noise and interferences. For this to be conducted, adaptative bit rates and data redundancy are employed, in order to adjust the robustness of the transmission to the environmental conditions. Besides, many LPWAN technologies employ sub-GHz frequency bands, hence obtaining longer transmission distances and wave penetration in walls than those solutions employing the 2.4 GHz bands, such as WiFi of BLE. This is of relevant importance for wearables, as they may operate in different indoor and outdoor scenarios during a normal day. In addition, the use of lower transmission frequencies permits electronic circuits to be more energy efficient, which is another desired characteristic, as stated above.

In the light of these features, LPWAN-based solutions seem to be highly convenient for wearable devices, although some additional aspects should be considered as well. Most LPWAN technologies make use of the industrial, scientific, and medical (ISM) frequency bands, which are free and open to be exploited. For this reason, they are highly saturated in certain areas. In order to control the use of these bands, a strict duty cycle is established by the corresponding regulation bodies; for example, in Europe, each node is allowed to transmit just 1% of time per day. This notably restricts the number of transmissions per device, although this limit is usually enough for most IoT applications. This issue is overcome by certain LPWAN solutions, by employing licensed spectrum, at the expense of introducing additional costs. LPWAN’s long transmission distances are achieved by reducing the transmission data rate to the order of kilobits per second (kbps). This fact prevents end-devices to exchange heavy data, such as multimedia content. Again, considering the potential applications of wearables, the produced data streams should not be continuous and with a limited bandwidth, therefore assumable by most LPWAN technologies. Besides, as discussed later, novel optimized ML techniques will permit a reduction in the communication activities of end-devices, as the transmission of raw data to the cloud will be strictly limited.

Currently, the most relevant LPWAN solutions are LoRaWAN, Sigfox, and narrow-band IoT (NB-IoT), which are notably different in their concepts and infrastructure designs [8]. Sigfox and NB-IoT bet on an infrastructure that is managed by a telco operator and, while Sigfox is an independent and standalone solution, NB-IoT is linked to the further development of the 4G and 5G networks. In turn, in a first instance, LoRaWAN proposes the deployment of private networks that should be maintained by their owners, although it may also be purchased from diverse commercial providers, which eases the deployment and management processes (https://www.semtech.com/lora/ecosystem/networks; accessed on date: 23 July 2021). This leads to two different models that may fit into different use cases. Sigfox or NB-IoT requires a monthly subscription to gain connectivity, as it leverages on an already deployed and managed infrastructure that makes use of licensed frequency bands, hence avoiding channel saturation issues, as aforementioned. This is an adequate alternative when a plug-and-play solution is desired; however, the expansion of this infrastructure is subject to the telco decision. On the other hand, the model adopted by LoRaWAN permits the vast deployment of networks that are freely accessible, but are not supported by a big company. A clear example of a wide and international LoRaWAN deployment is The Things Network (https://www.thethingsnetwork.org/; accessed on date 23 July 2021). In line with this, many city councils are deploying LoRaWAN networks as part of their plans to transform current urban settings into smart cities [10].

From the perspective of wearables, the use of both technologies is complementary, as they will coexist in future connected scenarios. While NB-IoT permits greater transmission rates and good coverage in urban areas, LoRaWAN is more cost and power-consumption effective, and presents the possibility of a quick coverage extension with a limited investment [8]. The advantages of integrating LPWAN communication modules within wearables are clear, given their low price, very low-power consumption and the possibility of detaching the device from the smart phone. Thereby, wearables will be able to bidirectionally communicate with the cloud, for example, adopting the IPv6 addressing scheme [11], which is greatly attractive for the development of novel services and applications. Besides, LPWAN-based device-to-device solutions will also allow the direct communication among end-devices, which is highly relevant in the case of developing distributed intelligent systems, as explored below.

### 2.2. Embedded Intelligence: ML-Based Mechanisms

Different ML-related solutions have recently emerged, aiming at converting IoT systems and devices into smart entities. In line with this, both on-device and distributed intelligent mechanisms are considered as two different approaches for reaching this goal.

#### 2.2.1. On-Device Intelligent Mechanisms

**Figure 1:**
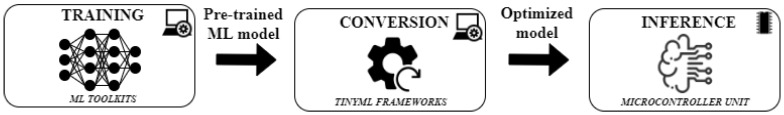
TinyML workflow.

As mentioned previously, TinyML is a recently arrived paradigm that permits the embedding of powerful ML models in resource-constrained units, such as wearable devices [12]. The usual TinyML workflow is presented in Figure 1. As can be observed, it is split into three well-differentiated phases. In the first one, the selected ML algorithm is trained in a computer or server, in order to obtain a regular ML model. To this end, typical ML frameworks can be employed, e.g., scikit-learn, Pytorch, TensorFlow, Keras, etc. The obtained model is then optimized and ported to a coding language that is supported by constrained end-devices, normally C in some of its versions. This task is performed by different TinyML toolkits from both big companies (e.g., Google, Microsoft, ARM, etc.) and developers that can be used without cost in some cases. A detailed survey of the TinyML frameworks that are available in the market can be found in [6]. The output of the conversion stage is a TinyML model that has inherited all the properties of the original one, but it is optimized to be embedded and executed by a microcontroller unit. Finally, in the last step, the ML-based inference is performed by the end-device, which now may be considered as a smart entity, leveraging the capabilities that are introduced by the integrated ML mechanism.

Focusing on the benefits of embedding TinyML-based intelligence within wearables, they are manifold. Firstly, as discussed previously, this enriched processing capacity will permit the detachment of the constrained unit from the master device. This is important in order to obtain truly autonomous devices that are able to make their own decisions. Besides, increasing on-device processing also permits the reduction in communication tasks. Although in the previous section the LPWAN technology has been identified as an energy-efficient alternative in comparison with other communication solutions, it is still more consuming than moderate local processing. Regarding the delay in obtaining the output of a certain computation, the process of sending data to the cloud and waiting to receive the answer is notably slower than performing the data processing locally. This aspect is crucial for non-delay-tolerant applications. Furthermore, considering that many applications of wearables are oriented to monitor fitness or health metrics, the privacy and security of these data should be ensured. For that reason, conducting on-device processing of the collected data avoids possible data leaking and other cybersecurity risks [13]. Besides, current wearable devices lack a high grade or personalization. This is important for tailoring the experience that is offered to each user, in an automatic way. User and gesture recognition, emotion analysis, etc., will permit the development of wearable applications that are capable of dynamically adapting themselves to the state of the user in real time.

In light of this discussion, it can be concluded that the dependency of wearable devices from the cloud can be notably limited, thanks to the TinyML paradigm. Besides, the reduction in communications with the cloud shortens task latencies at the time of improving data security and user experience. However, the main limitation of the strategy followed by TinyML is that the optimized models are hard to be updated once embedded in the device. Therefore, they are static in nature and cannot be updated on-the-fly. To cope with this issue, other AI-distributed solutions have also recently emerged, with the aim of permitting end-devices to build ML models in a cooperative and dynamic way.

#### 2.2.2. Distributed Intelligent Mechanisms

**Figure 2:**
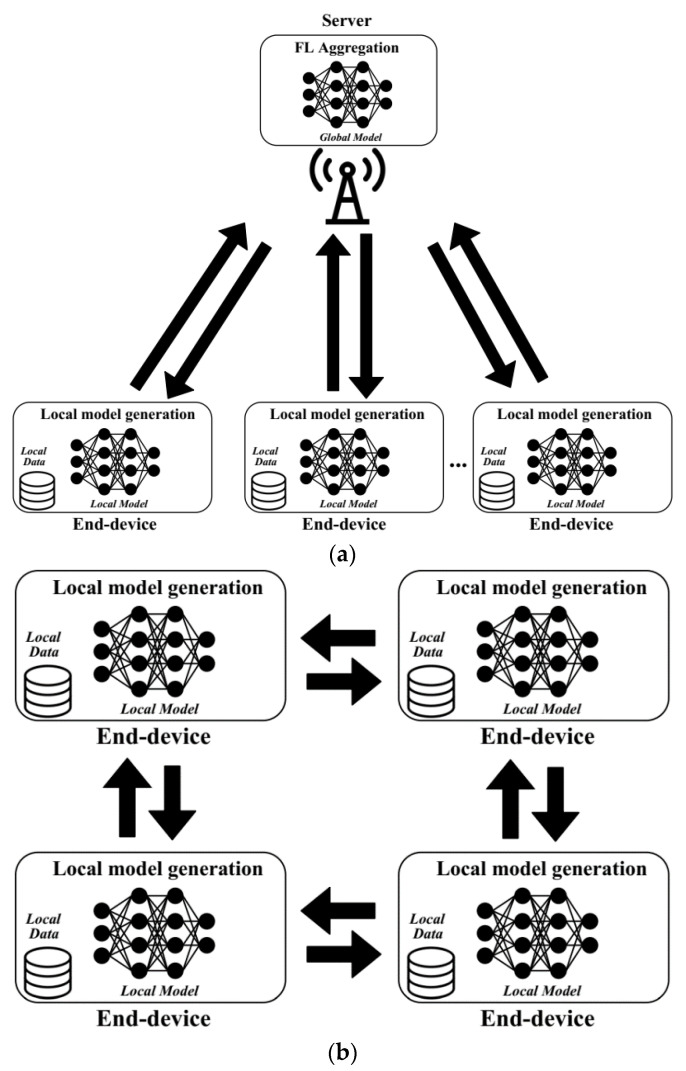
Federated learning (FL) models: (**a**) centralized architecture, (**b**) decentralized architecture.

Given the mobile nature of wearable devices, and their vast number in the near future (together with other IoT devices), in some applications it can be highly beneficial to train and build ML models from decentralized data, and in a distributed way. This is the proposal of the federated learning (FL) paradigm. Concretely, FL permits the coordination of a number of end-devices with a central server for collaboratively building a global model using the local data of each end-device, although without sharing these raw datasets. To this end, firstly the central server builds the first version of the global model, with certain initial parameters. Then, each end-device downloads the model and updates it locally by using its own dataset. Once the model has been updated, it is uploaded back to the central server, which aggregates and combines all the model updates that were received from the end-devices. The model generation may be conducted in different ways. For example, the whole model may be cooperatively built by all end-devices, or each end-device (or a group of them) may be in charge of building a certain portion of the whole model. After some iterations, the model will be refined, thanks to the collaboration of the constrained units, which will finally obtain an updated and rich ML model that has been built with a variety of local datasets of each end-device. In addition to this approach, based on the coordination of end-devices from a central point, there is another decentralized strategy that permits end-devices to coordinate themselves without an external entity. Thus, IoT units communicate with each other in a p2p fashion, and, following a similar process as that described above, after a certain rounds of model exchanges and updates, they obtain a finally consensual model [7]. Each of the mentioned strategies, centralized and decentralized, are related to different networking architectures, which follow different paradigms, such as edge [14] or fog [15] computing, respectively. Figure 2 presents the architecture of both the approaches.

Following the FL strategy, a number of benefits are brought to IoT devices in general, and wearables in particular. Firstly, the model quality is improved, as it is trained using different datasets, hence expanding its training inputs. This is of crucial importance for a wearable unit, given its dynamic nature, as it will be ready to adequately react to situations never seen before by itself, but already captured in others’ datasets. Besides, this learning process will be maintained during the system’s lifetime, as the datasets will be continuously updated with new end-devices’ observations. This will permit constrained units to get smarter over time, which is different to the TinyML solution that, as aforementioned, is more static, although the firmware over-the-air (FOTA) concept may fill this gap and permit TinyML models to be dynamically updated as well [16].

Similar to the TinyML approach, FL will allow the reduction in communication tasks in comparison with strategies based on centralized processing in a datacenter. Observe that no raw data is transmitted from the end-devices to the central server, but just the ML model parameters. This has two main implications. Firstly, the volume of transmitted data is notably limited, with the energetic benefits discussed previously. Besides, considering the low data rate of LPWANs, as the amount of exchanged data is decreased, the latency on the model generation is also reduced. Secondly, data security and privacy are also enhanced, as the transmission just includes model parameters, which are difficult to understand by a malicious observer. This is crucial in the case of wearables, as they usually handle sensitive data from the carrier, such as health metrics or his/her geographical position in real time.

Another important feature of FL is the transparent interaction of several types of devices. Firstly, these elements may be working in different environments, thus enriching the finally produced model. This will permit wearables to host intelligent mechanisms built by themselves and by other gadgets, hence expanding their possibilities and capability to react against situations they have never seen before. In addition, the use of different datasets also prevents the obtention of biased models, which may be limited by the data captured by a specific class of device.

The integration of intelligent algorithms in resource-constrained units is not something new [17]. However, the proposals that can be found in the literature are ad hoc solutions to deal with specific problems or use cases. The development of well-defined methodologies and procedures for embedding intelligence within end-devices is a step forward for extending the use of solid ML libraries, e.g., scikit-learn or TensorFlow, instead of fostering individual efforts with limited generalization capability. This will fuel the integration of reliable ML mechanisms in a plethora of IoT and wearable units, with all the advantages identified above. Consequently, and given the open nature of many TinyML toolkits, a rich and dynamic ecosystem of researchers, developers, and makers will be created.

To conclude this section, it can be observed how the recent advances in both communication and AI technologies will provide a great momentum to the wearable industry. The reviewed solutions provide novel capabilities to end-devices for interacting with the environment, the cloud, and other IoT elements, in a smart and context-aware way. Undoubtedly, this paves the way for the development of innovative services and applications, as discussed in the following.

## 3. Discussion: The Future of Wearables

The LPWAN and intelligent optimized mechanisms analyzed above are the building blocks for future wearable devices and applications. As discussed previously, they will permit the definitive detachment of end-devices from the smartphone. This is important in several use cases, where removing this heavy and bulky element is a clear benefit for the user, such as in the following: jogging, water sports, elderly people monitoring, etc. Besides, in order to increment the autonomy of wearables, battery-free solutions are also being investigated [18]. In the following, insights regarding the wearables applications are provided, which will be potentially benefited from the integration of long-range communications and intelligent capabilities.

### 3.1. Evolved Wearables Applications

The sectors within the wearables ecosystem that will be benefited from the integration of the technologies under consideration in this paper are multiple. In the first place, the most popular wearable devices, i.e., fitness and activity trackers, will increment their features and services offered to users, e.g., earbuds for monitoring vital signs [19]. Given the variety of physiological and biomechanics metrics that they are able to measure, tailored monitoring for each specific user will be enabled, thanks to the integration of intelligent processing. This is of relevant importance for professional athletes with the aim of meticulously planning their training sessions to maximize their performance [20]. Intelligent wearables will become advanced personal trainers, by means of human activity recognition (HAR) mechanisms [21], hence being capable of analyzing the athlete posture and dynamics. In line with this, the device will be able to flexibly readjust its behavior depending the user and contextual circumstances, e.g., scenario, climatic conditions, user status, etc. Thus, thanks to the wearable’s embedded ML models, precise medical diagnosis, improved physical rehabilitation efficiency, or detailed sport performance analysis, among others, are services that will be available in the next generation of fitness and sport trackers. Besides, the integration of long-range transmission capabilities will enable the quick triggering of alerts in the case of an emergency, such as in mountain and water sports, or activities in remote areas, in general. Other interesting solutions are oriented to improve the safety of pedestrians and joggers, by warning them when a vehicle is approaching [22].

Clearly, the eHealth industry is another hot niche for the development of innovative applications, thanks to the upcoming smart wearables, specially implanted devices, e-patches, or e-tattoos [23]. Prompt disease detection is fundamental for the diagnosis of illnesses before their first symptoms [24]. In line with this, a quick detection of infectious diseases is crucial in order to avoid pandemics [25]. Besides, having continuous monitoring of critical patients [26], chronic diseases [27], or elderly people [28], is greatly relevant for the patient’s quality of life. This monitoring is usually conducted in indoor environments, hence the exploitation of LPWANs for maintaining periodic contact or triggering alerts to the patient’s healthcare service may be highly beneficial [29]. Considering mental disorders, emotional recognition is critical for providing an adequate care service and ensuring the patient’s well-being [30]. Advanced wearables for this kind of patient, or others with physical disabilities, would be highly valued, such as automatic drug dispensers [31] or personal assistants, e.g., the smart-belt for blind people, recently proposed in [32]. Regarding the latter, end-devices with natural language processing [33], computer vision [34], or city navigation [35] capabilities, will be essential to increase the autonomy of impaired people. Smart jewelry and straps are other devices that are currently receiving great attention, given their features to be employed in a range of eHealth-related services [36]. 

Personal assistants may be oriented to other multiple applications in both personal and industrial segments. They will enable an enriched user experience through the exploitation of augmented reality mechanisms. For example, smart glasses and masks will be employed in industrial environments to help workers in their daily tasks, for example, by guiding them through a factory, indicating the precise point of failure in a complex system, or providing virtualized controllers [37,38]. In this case, the independence of the wearable unit from a master device will allow the freedom of both hands, which is highly important in this kind of scenario. Aligned with the natural language processing use case identified above, smart hearables will be capable of translating incoming speech in real-time, thus improving the interaction between people that do not share a common language. Regarding this interaction between individuals, the detection of other’s sentiments and mood will help to better understand each other [39]. Finally, eyerables, or smart glasses, will enable advanced infotainment services in different domains, by means of virtual reality. For example, tourists will receive updated city maps or real-time information of the monuments they are visiting [40]. In turn, intelligent earables, such as headsets or earbuds, will be able to automatically select the most adequate music according to the emotional status of the user [41].

The market of smart clothes and e-textiles has also notably been growing during the recent years, and it is predicted that their penetration will increase in the following years [42]. The integration of intelligence in garments will enable the active sensing of biometric information, aiming at making them adaptive to the user conditions. Clear examples are continuous body temperature sensing and regulation [43], or detection of excessive sun exposure [44,45]. Intelligent clothes will boost the development of multiple applications in sensitive ambits, such as the baby’s heart and breathing rate monitor presented in [46], which triggers an alert in the case of detecting anomalies. Besides the fitness wearable gadgets mentioned above, a new wave of intelligent textiles will be oriented to this market segment. Considering professional athletes, smart clothes will be able to intelligently track their activity for providing specialized support for reaching certain goals. This concept, known as wearable coach, is being deeply investigated, and some authors have proposed to include built-in haptic vibrations for pulsing particular body parts, with the aim of encouraging the athlete to move it or hold a certain posture [47]. As mentioned previously, wearables will have an important role in the improvement of rehabilitation processes [48]. Another hot research line is the use of e-textiles for ambient sensing, in order to develop intelligent ambient-assisted living applications for elderly people [49], or environmental monitors for detecting harmful levels of pollution [50]. Given the large size of certain garments, e.g., shirts or pants, they can be employed as smart energy harvesters, by means of flexible solar panels to provide energy to other wearables [51].

Finally, the smart interaction of wearables with other gadgets will pave the way for the development of the tactile and gesture-based internet [52]. In addition, gesture- and touch-sensitive clothes will permit the easily control of applications in other screens, e.g., maps or multimedia services, as proposed by Samsung’s smart suit (https://samsung.com/; accessed on date: 5 July 2021) or Google’s Jacquard (https://atap.google.com/jacquard/; accessed on date: 8 July 2021) projects.

### 3.2. Challenges

Although the development of the next generation of wearables keeps progressing at a good pace, there are still certain challenges that need to be addressed in order to achieve the successful integration of the intelligent and communication technologies that have been reviewed above.

#### 3.2.1. Energy Constraints

Firstly, the most important challenge to overcome is the energetic limitations of wearable units. Although the combination of LPWANs and embedded ML permits the reduction in the volume of the power-demanding communication tasks, the small size of wearable’s batteries prevents them from providing large capacities, and, consequently, long working times under intense operations. Wireless charging is offering more flexibility and convenience to the users; however, charging devices is usually annoying, hence its frequency should be reduced. Batteries are being improved in their efficiency and size, and, even though the most employed element is still lithium/lithium-ion, other innovative materials, such as graphene, are also being investigated [53].

However, the related field of study that is receiving more attention during the recent years is energy harvesting. As mentioned previously, energy harvesting is a promising technology for wearables, as it will permit the charging of batteries on-the-fly, making these devices completely autonomous from the power grid. The sources of energy under study are manifold, with kinetic, thermoelectric, and solar energy harvesting being the most advanced ones. The former is a highly interesting option for wearables, as it exploits the energy generated by the motion of the device carrier. Many studies have focused on the foot as the most convenient body part for placing this type of harvester [54], and some self-sustainable simple wearable devices based on inertial kinetic energy have been already proposed [55]. On the other hand, thermoelectric energy harvesting converts the temperature gradient between the human body and the environment into electric energy. This option is especially oriented to devices with certain surface in touch with the skin, such as smart watches, e-patches, or e-shirts, among others. Leveraging this technology, the first battery-free body-powered prototypes have been developed, as reported in [56]. Solar and ambient light energy harvesting is the most common and advanced energy harvesting technology. In line with this, watches and shirts are the most employed form factors for solar-powered devices. As aforementioned, the latter is a convenient solution, given the dimensions of the solar panel, which may permit energy to be provided to other devices worn by the user [57]. Finally, it is worthy to mention another energy harvesting methodology that is gaining momentum, consisting of converting the energy of ambient radio-frequency waves into electric energy. This technology leverages the electromagnetic energy of the omnipresent radiocommunication networks, such as WiFi, BT, or cellular solutions. Although the energy conversion rate is still very low, interesting advances are being achieved in this field [58,59].

#### 3.2.2. Computing Limitations and Device Heterogeneity

Given the energy constraints mentioned above, wearable devices usually mount efficient microcontrollers as processing units. Although notable advances are being conducted in the computation power of these elements, they are still far from modern CPUs. As discussed in the previous sections, the recent dawn of the TinyML paradigm paves the way for enabling complex ML-based processing within the device. This is crucial for the development of novel smart services and applications provided by the wearable device, without the support of external entities. Besides, it should be considered that wearable units have very specific functions, hence the scope of their implemented activities is noticeably narrow, so the processing algorithms can be highly optimized for these specific tasks. However, some heavy computing tasks are not adequate to be executed by the device, so they should be offloaded. Great efforts are being devoted to the computation offloading paradigm, especially aligned with the edge computing concept [60,61]. As communication activities should be limited, given their notable energy consumption, the amount of data exchanged with the edge or cloud should be reduced. At this point, it is important to mention the data aggregation solutions that are being developed, leveraging the power of diverse ML-based mechanisms [62]. 

In addition to on-device computation and task offloading solutions, distributed approaches, such as FL, open a variety of possibilities to share processing resources that are available in other cooperative constrained units. The main advances in this field have been achieved under the umbrella of the fog computing paradigm [63]. The main challenges of this computation model are related to the correct coordination of participants, and the need to be greatly efficient in both computation and communication tasks for reducing energy consumption. However, as explained previously, the fact that each device may cooperate with its own data and contextual knowledge is greatly beneficial for all the participants, as the obtained outcomes are not limited to the scope of a single element. This strategy permits the aggregation of small portions of available computing resources in different devices, hence building a web of collective intelligence.

Regarding the variety of wearable devices that are already available on the market, as well as the new ones that will appear in a short amount of time, it is necessary to find a common ground for the straight and fruitful integration of the computation and communication technologies treated in this article. The main issues are related to the former, because, in the case of embedding intelligent algorithms, the computing and memory resources of the device dramatically determine the complexity of the integrated mechanisms, as explored in Section 4. The large range of hardware platforms in the market is a non-negligible obstacle for producing a set of embedded ML frameworks that could deal with the constraints of most microcontroller-based units. Besides, this also is an impediment for designing agnostic benchmarking methodologies and toolkits [64]. For those reasons, the possibility enabled by the TinyML paradigm of employing well-known and general-purpose ML libraries for developing the desired optimized models is crucial for obtaining solutions with great generalization capability, which will increase the awareness and adoption of the developed solutions. Finally, with regard to the issues of integrating different transmission technologies within the wearable devices, they are usually related to the dimensions of the communication interface, and, specially, of the antenna. The size of the antenna is an important factor, as it determines its transmission and reception gain. Thus, employing communication technologies and modules with very high sensitivity, such as LPWANs [4], will allow the dimensions of the involved peripherals to be reduced, therefore easing their integration within the wearable unit.

#### 3.2.3. Data Security

Last, but not least, the confidentiality and integrity of the data that are wirelessly exchanged by the wearable, with the infrastructure or other devices, must be ensured. This is of prominent importance, considering the private information that these data may contain. Focusing on data confidentiality, the cryptographic algorithms should be strong enough against different types of attacks, such as eavesdropping, traffic analysis, etc. These threats may increase, considering that the end-devices will gain direct connectivity with the internet, for example, by receiving a public IPv6 address [65]. The limited processing and memory resources that are available in wearables make the implementation of highly robust encryption schemes and other defensive schemes difficult, as they would be greatly time and energy consuming. This is especially critical in the case of using LPWANs, given their constraints in the amount and size of the transported messages. Different solutions are under study, to enable efficient key generation and renewal for constrained end-devices [66], even being compliant with the security requirements of the novel 5G architecture [67]. The malicious access to the data that are transmitted by a wearable may permit user identification, as well as exposing sensitive data, which easily allows the monitoring user habits and real-time sensed information. For example, sophisticated analysis of the data generated by a smart watch permits the inference of user inputs to keyboards, with the aim of revealing passwords [68,69]. Many other vulnerabilities and cyberthreats affect wearables [70], hence novel solutions, for example, based on embedded ML [71], which may permit the dynamic adaptation of the device to future cyberattacks, would be highly desirable.

Considering data integrity, it is also of prominent importance to avoid the transmitted information being altered in transit or the user identity being supplanted. Regarding the latter, impersonation attacks are critical in different applications, such as those related to medical or financial services. They may permit the attacker to not just supplant the user identity, but also gain access to the network or data infrastructures [72]. To cope with these issues, the development of mutual authentication methods, i.e., end-device network, is a valid solution. However, they usually rely on asymmetric cryptography or digital certificates, which require great computational and memory resources. Recent proposals, such as the one in [73], allows a secure and efficient bootstrapping of constrained IoT devices that are connected to different kinds of network infrastructures. The bootstrapping process is of prominent importance for ensuring the transactions of the device with the network. It requires a range of security operations, such as authentication, authorization, and key management, which should be tailored to the communication and processing capabilities of the end-device. Thus, once authenticated, the wearable unit may exchange data with remote servers or other surrounding devices in a safe way. Wearable device authentication is a hot research field, and different solutions are being proposed according to the requirements of the different families of wearables [74]. 

## 4. Use case

This section aims to propose a general device architecture for next-generation wearables, as well as presenting the results of a real implementation in which the following have been explored: (i) the connectivity gained by a wearable unit through an LPWAN deployment, and (ii) the supported complexity of certain ML mechanisms embedded in the device. 

### 4.1. Device Architecture

**Figure 3:**
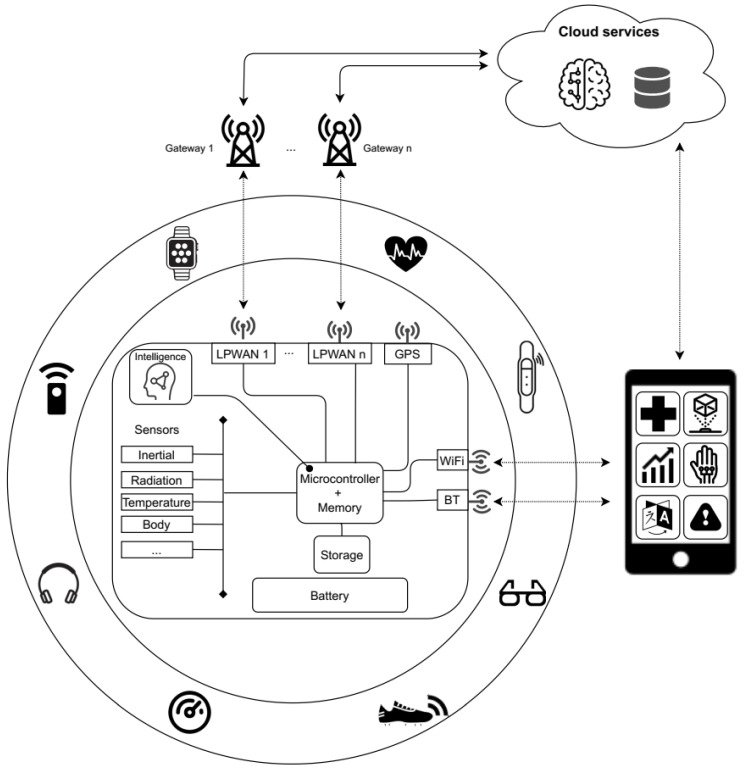
Wearable device architecture.

A general architecture for wearable devices, integrating the communication and AI technologies explored previously, is presented in Figure 3. As can be observed, its central entity is the processing unit, which consists of a microcontroller, as well as the required program memory space. Here, the main limitations of the device can be found, as this element should be highly energy efficient, cost effective, and with a reduced size. Considering the need of storing a certain amount of sensed data for on-device ML processing, a storage bank is also necessary, although this is not a limiting aspect, given the novel generation of tiny storage solutions. As depicted in the diagram, a series of sensors (or even actuators) and a GPS module are also integrated within the unit. The nature of the former will depend on the target application, and they should also be highly power efficient during their data sampling activities. Regarding the energetic aspects, the battery is another key component of the device, as it should be large enough to provide long working times, but its weight and dimensions should certainly be limited. As discussed previously, some recent proposals are evaluating the performance of energy harvesting systems that are attached to wearable devices [56]. 

Regarding the integration of intelligent mechanisms following some of the paradigms discussed before, i.e., TinyML or FL, they should be meticulously adapted to the target unit, considering its computing and memory constraints. To give insights about this issue, in the following sections, a detailed study addressing the limitations of a well-known microcontroller unit is presented. As will be discussed, the integration of the optimized ML model may present issues that are related to the limitations of program or data memories, depending on the algorithm implementation. The target device will also be crucial for determining the complexity of the ML-based model. As can be observed in the figure, the intelligent system may be integrated in a plethora of devices with different requirements and needs, in terms of input data, response latency, etc. Thus, the embedded intelligence will interact with the stored data in order to make smart decisions (TinyML) that may trigger certain actions or contribute to the cooperative development of an FL-based model, among other activities [6]. 

Considering communication aspects, given their low energy consumption and the opened possibilities identified above, a number of LPWAN interfaces may be integrated into the unit. They will be able to directly interact with cloud services that will complement on-device tasks by providing extra applications or nicely presenting information, and report to the user. To this end, he/she will make use of personal devices, such smartphones or tablets, to access and visualize the data by means of specialized apps or web-based dashboards. Given that the connectivity between the wearable unit and this kind of device will still be necessary, additional communication interfaces, such as WiFi or Bluetooth, will be present, although their use will be notably reduced, as explained in the previous sections.

### 4.2. Implementation

**Figure 4:**
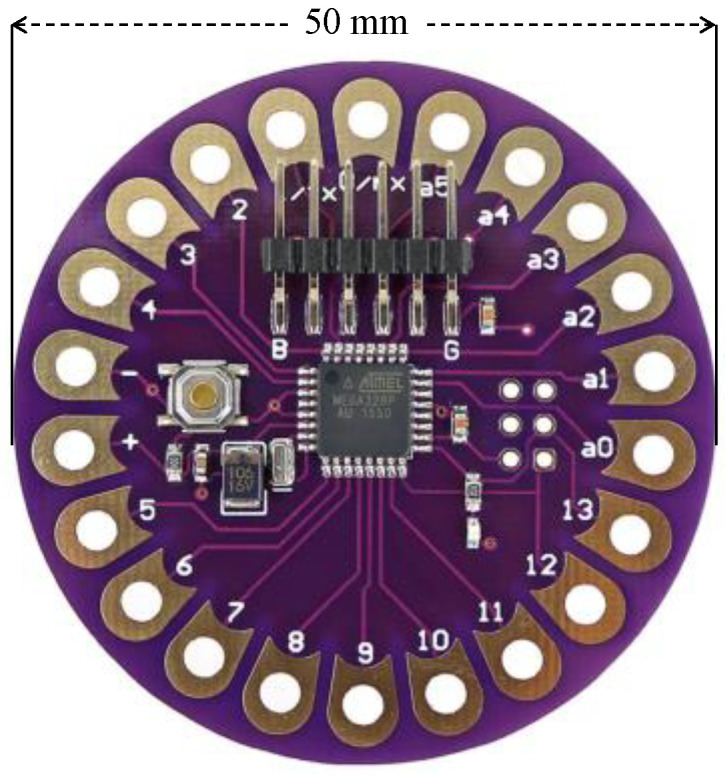
Wearable unit employed in the experiments.

Aiming at studying the performance of a real wearable device, integrating the technologies discussed in the paper, a functional wearable unit has been implemented. To this end, the wearable device that is shown in Figure 4 has been selected. The reason for choosing this unit is that it is powered by the ATmega 328p microcontroller, which is widely known, as it is integrated within the Arduino Uno board. This microcontroller is a highly restricted solution, as it presents an 8-bit processor at 16 MHz, and with flash and Static RAM (SRAM) memories of 32 and 2 kB, respectively. 

Regarding the connected peripherals, the GY-NEO6MV2 GPS module has been used, employing the Arduino’s TinyGPS library for interacting with it. Besides, the wearable unit has been provided with LPWAN connectivity, by means of the RN2483 LoRaWAN module and a 2dBi helical antenna attached to it. This chip is a certified LoRaWAN device, and is very popular in both academia and industry implementations, as it provides a well-documented and easy-to-use library. The communication with both modules are conducted via two virtual serial ports, opened thanks to the Arduino’s SoftwareSerial library. Finally, the MPU6050 accelerometer and gyroscope sensor has been connected to the wearable unit as an example of the possible embedded sensor.

Both the MPU6050 and Arduino’s Wire libraries have been used to communicate with this module via an I2C (Inter-Integrated Circuits) connection. The specification of the libraries that were employed in our implementation is important, in order to analyze their memory footprints, which certainly determine the maximum complexity of the intelligent algorithm to be integrated. Finally, a 2000 mAh powerbank was employed to power the built solution.

### 4.3. Experiment Details

**Figure 5:**
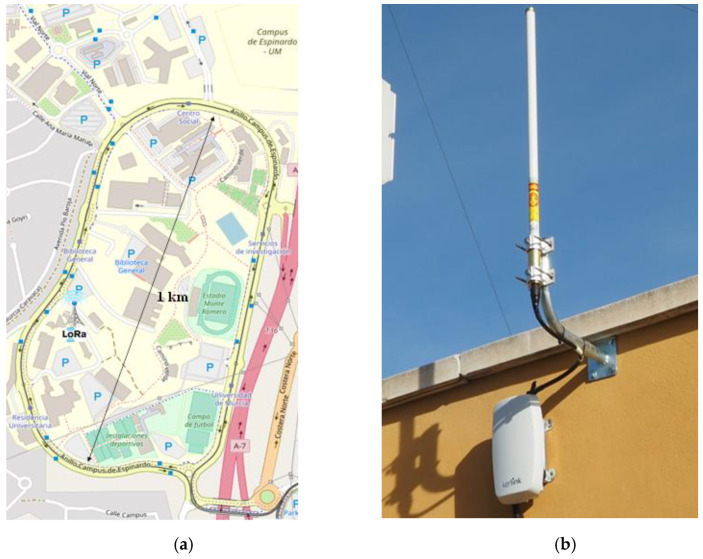
LoRaWAN deployment: (**a**) deployment map; (**b**) LoRaWAN gateway detail.

As explained previously, two different studies have been conducted. Firstly, a coverage test has been conducted in a university campus scenario, with an already deployed LoRaWAN infrastructure. This study has consisted of a sampling campaign along the campus, in both outdoor and indoor locations. Concretely, a ping application has been developed in order to test the connectivity of the device with the infrastructure, in both uplink and downlink directions. As shown in Figure 5a, the exploited LoRaWAN infrastructure consists of one LoRaWAN base station placed within the Espinardo Campus of the University of Murcia (Spain). This element is a Kerlink LoRaWAN gateway, with an 8 dBi-gain antenna placed on the roof of a three-story building, at a height of approximately 10 m above ground level (Figure 5b). Regarding the end-device’s LoRaWAN configuration, the LoRa physical parameters have been set as follows: spreading factor (SF) = 12, coding rate (CR) = 4/5, and packet length = 50 B. The developed application periodically sent a “ping” packet to an app server placed in the cloud, which automatically responded back upon the reception of the “ping” message. With this setup, the packet delivery ratio (PDR) and coverage level of the wearable device along the campus have been measured.

Thereafter, the complexity of the TinyML models that can be embedded in the wearable device has also been studied. To this end, the emlearn (https://github.com/emlearn/emlearn/; accessed on date: 15 July 2021) library has been employed in order to convert a series of random forest (RF) and multi-layer perceptron (MLP) models, generated by the Python’s scikit-learn framework. The selection of the emlearn library has been due to its wide compatibility with different ML algorithms, and because it is capable of generating a C code that is runnable by 8-bit processors, which is not a typical characteristic of TinyML toolkits [6]. For training the models, a public dataset [75] that is oriented to the automatic identification of the wearable carrier has been employed. Please note that, although an inertial sensor has been integrated in the wearable unit for consistency purposes, the accuracy and performance of the generated models for this task are out of the scope of this work. As mentioned above, the generated TinyML models have been embedded together with the different libraries that are needed by the connected peripherals (GPS, LoRaWAN modem, and inertial sensor). This strategy has been followed in order to explore a realistic use case, in which several elements are connected and work over the wearable unit. 

### 4.4. Results

In the following, the outcomes of the conducted experiments are presented and discussed. Firstly, the coverage gained by the wearable device, through the LoRaWAN interface connected to it, has been evaluated. After that, the footprints of different TinyML models embedded in the device are explored, aiming at providing insights about how much ML model complexity can be supported by the wearable unit.

#### 4.4.1. LPWAN Communications

**Figure 6:**
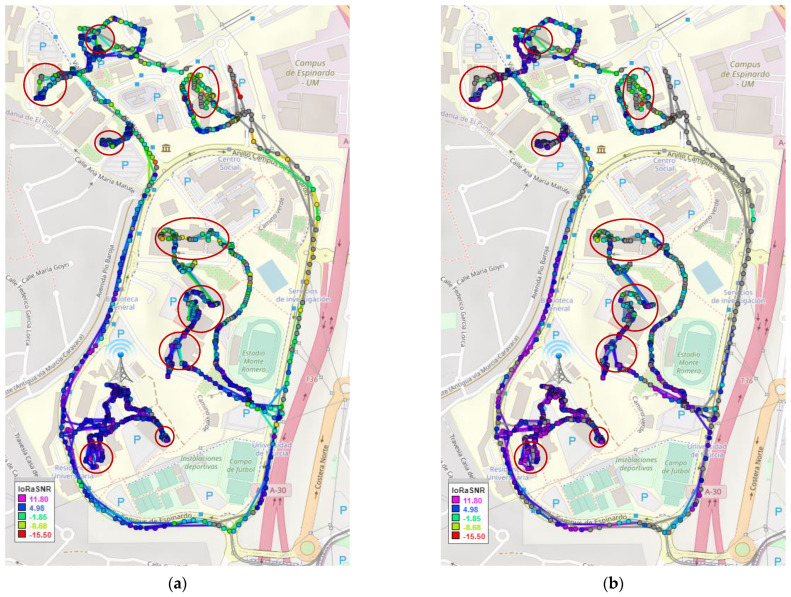
LoRaWAN SNR inside the campus: (**a**) uplink transmissions; (**b**) downlink transmissions.

As stated above, a sampling campaign has been carried out in order to evaluate the connectivity of the wearable within a university campus scenario, thanks to a pre-existent LoRaWAN deployment. This experiment was conducted by carrying the wearable unit while riding a bicycle or walking along the campus. Note that these measurements are not intended to comprehensively provide a performance evaluation of the LoRaWAN network in the campus, but to demonstrate the connectivity that a wearable device can gain, thanks to this kind of long-range low-power communication. Figure 6 shows the attained coverage and the signal level (signal-to-noise ratio (SNR)) in both the uplink (Figure 6a) and downlink directions (Figure 6b). Observe that the grey points indicate lost packets and that indoor locations have been indicated with red circles. In general, observe the good connectivity obtained by the wearable device along the campus scenario. The performance is notable in both the indoor and outdoor locations, which is a highly valuable characteristic for wearables. A coverage shadow has been found in the north-east area of the campus ring, as it is the most problematic zone, given the distance to the LoRaWAN gateway and the presence of dense vegetation and big buildings. This area introduces the major contribution to the overall packet loss obtained in the experiment. In order to provide more details about the reliability of the communications, Table 1 presents the packet delivery ratio (PDR) obtained in our trials. Concretely, it is studied depending on the type of location (indoor or outdoor) and the transmission direction (uplink or downlink). Observe the good figures obtained in all cases, always with a PDR over 92%. Although this is an expected result in outdoor environments [5], the good performance in indoor locations is remarkable. Besides, it can be observed that the performance is better in the uplink direction, which is the predominant link for this type of device. Given that the radio link was symmetric, this outcome can be explained by the better noise factor of the gateway radio module, as compared with that of the end-device. In light of these results, it can be observed how the wearable device is provided with long range connectivity with an efficient transmission technology.

#### 4.4.2. TinyML Integration

As mentioned previously, a series of ML models have been embedded in the wearable unit, by means of the emlearn TinyML framework. Concretely, the selected algorithms are as follows: random forest (RF), as a powerful generalization of the decision tree (DT) algorithm that provides great flexibility and generalization capability, and multi-layer perceptron (MLP), given the interesting possibilities and widely adopted neural network-based models. The integration of the produced TinyML models has been conducted together with the required libraries for the proper functioning of the peripherals connected to the wearable, namely, LoRaWAN transceiver, GPS module, and inertial sensor. This strategy allows the evaluation of the joint footprint of these libraries and the TinyML model on the device.

**Figure 7:**
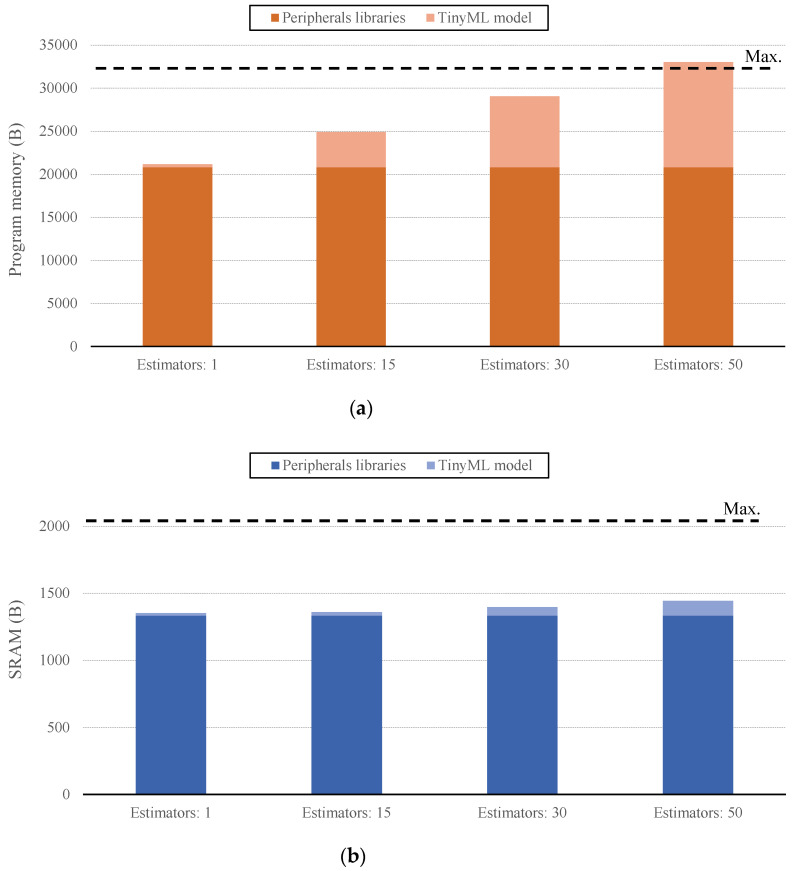
Memory footprint of the random forest (RF) TinyML implementation on the wearable device: (**a**) program memory; (**b**) static RAM (SRAM).

Focusing on the implemented RF models, some of their configuration parameters have been fixed as follows: criterion: gini; maximum depth: none; bootstrap: true; minimum samples to split: two. With these common parameters, a set of RF models with different internal estimators have been embedded on the wearable device. Figure 7 depicts the memory footprint on the wearable device of these models and the peripherals libraries. Concretely, Figure 7a presents the occupied program memory. As expected, the footprint of the peripherals libraries remains constant, and that of the RF TinyML model grows as its complexity is increased. As the RF’s nodes and branches are expanded, the generated code becomes heavier, which is directly reflected in the program memory occupancy. However, observe that a complex model, with 30 internal estimators, fits perfectly within the device. The maximum complexity of the RF model to fit within the wearable is about 50 internal estimators, which is a considerable figure that makes this model adaptable to different cases. In turn, observe, in Figure 7b, that the footprint on the SRAM is scarcely affected by the RF model. This is explained by the internal structure of the TinyML model implementation, which, in this case, does not make much use of variables that are stored in the SRAM. However, this behavior is not followed by the produced MLP TinyML models, as explained in the following.

The attained memory footprints for the produced MLP TinyML models are shown in Table 2 and Table 3. Given the number of evaluated models, this presentation format has been chosen for improving the outcomes readability. Observe that the performance of different neural network structures has been evaluated, considering several hidden layers and a number of neurons per layer. Regarding the impact on program memory (Table 1), observe that, differently from the RF models, the footprint on this memory is barely affected by the complexity of the MLP model. The total occupancy ranges from 72% to 74% of the available memory, depending on the number of hidden layers and neurons. However, it is clear that the impact of the TinyML model is much lower than that of the peripherals libraries. 

On the other hand, the major footprint of the MLP TinyML models is on the occupied SRAM, as shown in Table 2. In this case, observe its variation when the number of neurons in the network grows. The MLP configurations that surpass the maximum SRAM available in the unit are highlighted in bold. It can be observed that, in this case, the maximum number of neurons forming the neural network is about 60, which, again, is a notable figure that permits many MLP configurations. Differently from the RF models, in this case, the weights and biases of the MLP TinyML model are stored in the SRAM; therefore, this is the principal factor that limits the complexity of the embedded model.

The attained results suggest that the complexity of the models that can be embedded in the wearable unit may be enough to tackle an important range of use cases, as discussed in [76]. Even more, this complexity can be increased if the footprint of the other employed libraries is reduced. This is thanks to the good performance of the TinyML frameworks that are able to notably optimize and reduce the weight of complex ML models [6]. Besides, the use of LPWANs in this kind of device is also an interesting option that permits efficient long-range communications for detaching the wearable unit from its master device. Therefore, the confluence of LPWANs and embedded ML has the potential to be an exceptional opportunity for the wearable industry, as it may open the door for the development of new applications and services that have never been seen before [6]. Depending on the requirements of the application under consideration, the role of each of the addressed technologies will be different. Thus, although almost permanent connectivity may be achieved, through the exploitation of different LPWAN solutions, intelligent TinyML-based mechanisms can optimize network operations by means of advanced on-device processing. For example, local aggregation may be highly useful for performing adequate data curation prior to its transmission to the cloud. Furthermore, the coordination of distributed embedded ML models may lead to the cooperative development of intelligent mechanisms, which is a promising solution for obtaining flexible and non-static wearable devices.

## 5. Conclusions

This article has treated novel technologies that may boost the innovations in the wearables sector in the following years. Concretely, the focus was on the LPWAN and embedded ML paradigms. In both the cases, they fill important gaps that prevent current wearable devices to be truly autonomous and independent units from both communication and processing perspectives. Regarding LPWANs, they enable long-range transmissions with limited energy consumption, which permits end-devices to directly connect to the network infrastructure without using another paired element as a relay, e.g., a smartphone. On the other hand, the adoption of embedded ML solutions, such as TinyML or FL, allows wearable units to make their own decisions, without the support of the cloud or a master device. Thereby, this paper has focused on the integration of these state-of-the-art communication and artificial intelligence technologies within the wearable’s ecosystem, hence enabling the development of truly autonomous and smart wearable devices. Both the approaches have been evaluated by means of a real experiment, in which the performance of a widely used wearable unit supporting LPWAN communications and TinyML-based computation, has been studied. The attained results suggest the validity of the proposal, as the wearable connectivity and processing capabilities can be notably and successfully enhanced. As future work, it is planned to design and build a fully integrated wearable prototype, efficiently gathering a set of peripherals for enabling it to be adaptable to a series of use cases, thanks to the integration of additional embedded ML models.

## Figures and Tables

**Table 1:** Packet delivery ratio (PDR) in indoor and outdoor locations.

	Uplink	Downlink
**Indoor**	92.7%	92.4%
**Outdoor**	97.8%	96.15%

**Table 2:** Memory footprint of the multi-layer perceptron (MLP) TinyML implementation on the wearable device.

**Layers\Neurons per Layer**	**2**	**5**	**10**	**15**
**1**	Peripherals: 20,812 B TinyML: 2490 B Total: 72.2%	Peripherals: 20,812 B TinyML: 2550 B Total: 72.4%	Peripherals: 20,812 B TinyML: 2650 B Total: 72.7%	Peripherals: 20,812 B TinyML: 2750 B Total: 73%
**2**	Peripherals: 20,812 B TinyML: 2514 B Total: 72.3%	Peripherals: 20,812 B TinyML: 2586 B Total: 72.5%	Peripherals: 20,812 B TinyML: 2706 B Total: 72.9%	Peripherals: 20,812 B TinyML: 2826 B Total: 73.2%
**3**	Peripherals: 20,812 B TinyML: 2538 B Total: 72.3%	Peripherals: 20,812 B TinyML: 2622 B Total: 72.5%	Peripherals: 20,812 B TinyML: 2762 B Total: 73%	Peripherals: 20,812 B TinyML: 2902 B Total: 73.5%
**4**	Peripherals: 20,812 B TinyML: 2562 B Total: 72.4%	Peripherals: 20,812 B TinyML: 2658 B Total: 72.7%	Peripherals: 20,812 B TinyML: 2818 B Total: 73.2%	Peripherals: 20,812 BTinyML: 2978 BTotal: 73.7%
**5**	Peripherals: 20,812 B TinyML: 2586 B Total: 72.5%	Peripherals: 20,812 B TinyML: 2694 B Total: 72.8%	Peripherals: 20,812 B TinyML: 2874 B Total: 73.4%	Peripherals: 20,812 B TinyML: 3054 B Total: 74%

**Table 3:** SRAM footprint on the wearable device.

**Layers\Neurons per Layer**	**2**	**5**	**10**	**15**
**1**	Peripherals: 1333 B TinyML: 162 B Total: 73%	Peripherals: 1333 B TinyML: 238 B Total: 76.7%	Peripherals: 1333 B TinyML: 378 B Total: 83.5%	Peripherals: 1333 B TinyML: 518 B Total: 90.4%
**2**	Peripherals: 1333 B TinyML: 186 B Total: 74.1%	Peripherals: 1333 B TinyML: 274 B Total: 78.4%	Peripherals: 1333 B TinyML: 424 B Total: 86.2%	Peripherals: 1333 B TinyML: 594 B Total: 94%
**3**	Peripherals: 1333 B TinyML: 210 B Total: 75.3%	Peripherals: 1333 B TinyML: 310 B Total: 80.2%	Peripherals: 1333 B TinyML: 490 B Total: 89%	Peripherals: 1333 B TinyML: 670 B Total: 97.8%
**4**	Peripherals: 1333 B TinyML: 242 B Total: 76.9%	Peripherals: 1333 B TinyML: 346 B Total: 81.9%	Peripherals: 1333 B TinyML:546 B Total: 91.7%	Peripherals: 1333 B TinyML: 746 B Total: 101.5%
**5**	Peripherals: 1333 B TinyML: 274 B Total: 78.4%	Peripherals: 1333 B TinyML: 382 B Total: 83.7%	Peripherals: 1333 B TinyML: 602 B Total: 94.4%	Peripherals: 1333 B TinyML: 822 B Total: 105.2%

## Data Availability

Not applicable.
